# 3-Phenylcoumarins as a Privileged Scaffold in Medicinal Chemistry: The Landmarks of the Past Decade

**DOI:** 10.3390/molecules26216755

**Published:** 2021-11-08

**Authors:** Maria J. Matos, Eugenio Uriarte, Lourdes Santana

**Affiliations:** 1 Centro de Investigação em Química da Universidade do Porto (CIQUP), Departamento de Química e Bioquímica, Faculdade de Ciências, Universidade do Porto, 4169-007 Porto, Portugal; 2 Departamento de Química Orgánica, Facultade de Farmacia, Universidade Santiago de Compostela, 15782 Santiago de Compostela, Spain; eugenio.uriarte@usc.es; 3 Instituto de Ciencias Químicas Aplicadas, Universidad Autónoma de Chile, Santiago 7500912, Chile

**Keywords:** 3-phenylcoumarins, synthetic pathways, pharmacological activity

## Abstract

3-Phenylcoumarins are a family of heterocyclic molecules that are widely used in both organic and medicinal chemistry. In this overview, research on this scaffold, since 2010, is included and discussed, focusing on aspects related to its natural origin, synthetic procedures and pharmacological applications. This review paper is based on the most relevant literature related to the role of 3-phenylcoumarins in the design of new drug candidates. The references presented in this review have been collected from multiple electronic databases, including SciFinder, Pubmed and Mendeley.

## 1. Introduction

**Figure 1:**
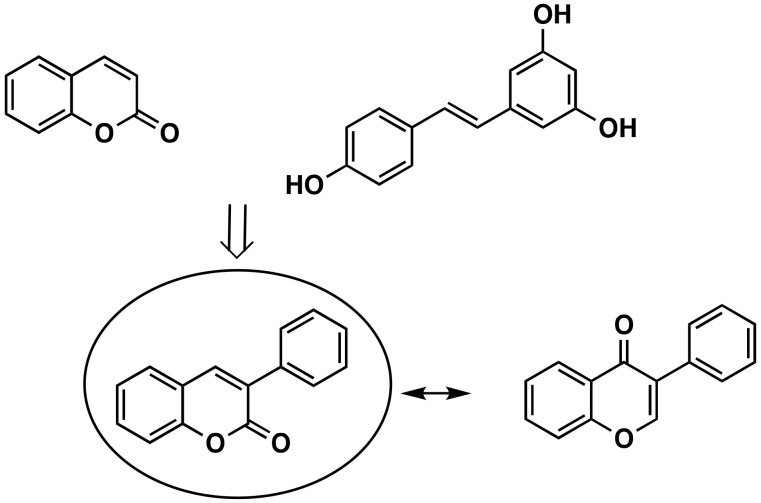
Coumarin, *trans*-resveratrol, 3-phenylcoumarin and isoflavone chemical structures.

3-Phenylcoumarins have been used by several research groups in the search for new chemical entities with potential in the discovery of new therapeutic solutions for several diseases. The versatility and chemical properties of this scaffold have been attracting the attention of researchers all over the world. Different molecular spots that may be modified, with different reactivities, allow for a huge number of different derivatives with different properties. This scaffold can be considered an isostere of the isoflavone in which the carbonyl group is translated from position 4 to position 2 on the pyran ring (Figure 1). Isoflavones are produced almost exclusively by the members of the bean family Fabaceae (Leguminosae). It can also be considered a coumarin-resveratrol hybrid. Resveratrol (3,5,4-trihydroxy-*trans*-stilbene) is a stilbenoid, a type of natural phenol, and a phytoalexin produced by several plants (Figure 1). The stilbenoids share most of their biosynthetic pathway with chalcones.

Coumarins, the basic structure of 3-phenylcoumarins, are a group of substances of natural or synthetic origin that are highly studied and have a great variety of pharmacological interests. In addition, a connection of 3-phenylcoumarins can also be established (although it is more from a structural or steric point of view) with steroid hormones, especially with estrogens, due to the aromatization of the A ring. For these reasons, 3-phenylcoumarins (Figure 1) are considered a privileged scaffold in medicinal chemistry. In fact, our research group is one of those that for many years has been studying this structure, preparing and evaluating analogues and derivatives of this chemical framework.

The current review is only focused on the 3-phenylcoumarins, without considering their analogues with associated rings or isosters. An extensive overview of the scientific literature since 2010 has been prepared, mainly focusing on aspects related to their natural origin, synthetic procedures and pharmacological applications. Only very brief references to their physicochemical properties or their application as fluorescence probes, a field in which references are quite abundant, have been included.

## 2. Presence of 3-Phenylcoumarins in Nature

**Figure 2:**
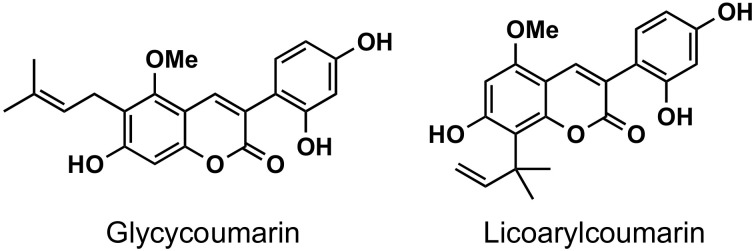
Chemical structures of glycycoumarin and licoarylcoumarin.

The naturally-occurring 3-phenylcoumarins that have been published in the past decade are listed in Table 1. Mucodianin A was isolated from the vine stems of *Mucuna birdwoodiana* [1]. The 3-(4-ethynylphenyl)-4-formylcoumarin has been isolated from a methanol extract of the red ants of ChangBai Mountain, *Tetramorium* sp. [2]. Pterosonin F was isolated from the heartwood of *Pterocarpus soyauxii* [3]. Sphenostylisin A was isolated from the root bark of *Sphenostylis marginata* using a bioactivity-guided isolation approach. It is worth highlighting that this compound is a potent NF-κB (nuclear factor kappa-light-chain-enhancer of activated B cells) inhibitor, displaying different physiological functions. This compound is also overexpressed in some cancer cells [4]. 2′,4′-Dinitro-3-phenylcoumarin was isolated from *Rhizophora mucronata* [5]. Selaginolide A was found in *Selaginella rolandi-principis* [6]. Glycycoumarin and licorylcoumarin (Figure 2) are two 3-phenylcoumarins previously isolated from *Glycyrrhiza uralensis* or *glabra* (Licorice), on which there are several studies in the past decade [7,8].

## 3. Synthesis of the 3-Phenylcoumarin Scaffold

There are multiple options for the synthesis of differently substituted coumarins. In particular, for the synthesis of 3-phenylcoumarins, three main methodologies have been used in the past decade: coumarin coupling with the benzene ring, construction of the coumarin bicyclic system and construction of the benzene ring at position 3. Papers including these reactions were analyzed, and the most relevant studies are described in the sub-sections bellow.

### 3.1. Coumarin Coupling with the Benzene Ring

**Figure 3:**
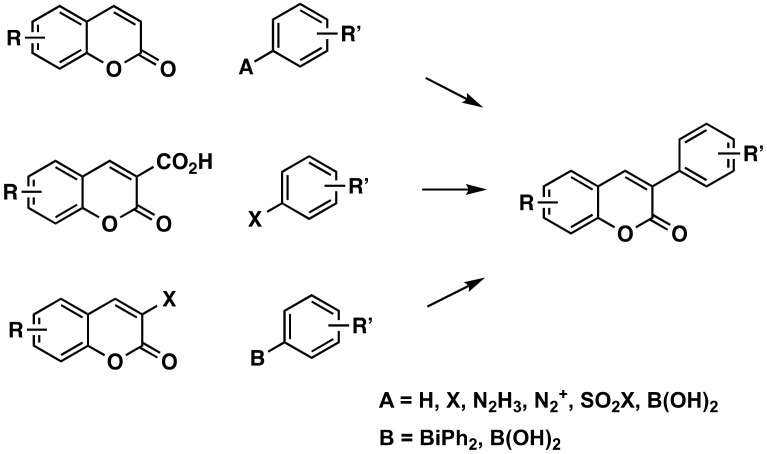
One of the synthetic routes to obtain 3-phenylcoumarins: coumarin coupling with the benzene ring.

Direct coupling of the benzene ring on the coumarin, at position 3, is one of the most common approaches to preparing 3-arylcoumarins (Figure 3). Different conditions, in particular different catalysts, have been used in the past decade. The information on these reactions is detailed in the next sub-sections, separated by the starting materials employed to achieve the final molecules.

#### 3.1.1. Without Prior Activation of the Coumarin and the Aryl

A regioselective α-arylation of coumarins can occur through an oxidative C-H/C-H cross-coupling between inactivated simple arenes and alkenes, with palladium acetate, in trifluoroacetic anhydride (TFAA), at 120 °C [9]. The coupling may also occur using highly electrophilic palladium species, with palladium(II) trifluoroacetate [Pd(TFA)_2_] as the catalyst, and cheap ammonium persulfate [(NH_4_)_2_S_2_O_8_] as the oxidant, under air, at room-temperature [10].

#### 3.1.2. Using an Activated Arene

Using an iodobenzene, a particularly useful, versatile and concise synthesis of differently substituted 3-arylcoumarins was achieved via Heck coupling reactions between coumarins and aryliodides using tetrakis(triphenylphosphine) palladium(0) [Pd(PPh_3_)_4_] as a catalyst [11]. This coupling reaction was also carried out on 4-hydroxycoumarins to obtain 3-aryl-4-hydroxycoumarins using, this time, palladium(II)acetate [Pd(OAc)_2_] as a catalyst [12]. The synthesis of 3-arylcoumarins can be performed regioselectively in moderate to good yields from oxidative arylation with phenylhydrazine, using potassium permanganate (KMnO_4_) as an oxidant [13], or directly, also with good yields, using potassium carbonate (K_2_CO_3_) at room temperature, under mild conditions [14]. There are examples of the synthesis of 3-arylcoumarins through a mild and environmentally friendly Meerwein arylation, using an aryl diazonium salt directly on the coumarin. A phenyldiazonium chloride, using cupric chloride (CuCl_2_) as a catalyst [15], or a tetrafluorborate, in the presence of catalytic amounts of 5,10,15,20-tetrakis (4-diethylaminophenyl)porphyrin [16], can be used. Recently, this last diazonium salt was used, mediated by chlorophyll as a biocatalyst, via visible light catalysis at room temperature, with good to excellent yields [17]. It was proven that a highly regio-controlled arylation of coumarins at C-3 was possible by using readily available arenesulfonyl chlorides and sodium arenesulfinates, via palladium-catalyzed, under mild reaction conditions [18]. An efficient KMnO_4_/AcOH-mediated dehydrogenative direct radical arylation of coumarins with arylboronic acids, to afford 3-arylcoumarin derivatives, has also been described. These coupling reactions occur in a moderate to good yield, and with a wide group tolerance [19]. The arylation at the 3-position of 4-hydroxycoumarins can be carried out in a simple and direct way, by photoinduced reaction with aryl iodides, in yields higher than 60% [20]. Very good yields were also obtained using a direct arylation strategy of 1,3-dicarbonyls with phenols that react at their *meta* position [21].

#### 3.1.3. From 3-Carboxycoumarins

Efficient and practical decarboxylative cross-coupling reactions of coumarin-3-carboxylic acids with aryl (or heteroaryl) halides (Br/I) under a bimetallic system of palladium(II)bromide (PdBr_2_) [22] or chloride (PdCl_2_) [23], and silver carbonate, have been performed in good to excellent yields. A highly regioselective and transition-metal free one-pot arylation of coumarins-3-carboxylic acid, in the presence of arylboronic acids, has been achieved, employing potassium persulfate (K_2_S_2_O_8_). The protocol consists in a sequence of some reactions, including an arylation/decarboxylation cascade, and proceeds properly in aqueous media with moderate to excellent yields [24].

#### 3.1.4. From 3-Halocoumarins

Cross-coupling reactions of functionalized 3-bromocoumarins with threefold arylating triarylbismuth reagent, in a palladium-catalyzed media to give functionalized 3-arylcoumarins in high yields, have been described. The authors described the procedure for obtaining 3,4-diarylcoumarins [25]. This reaction also occurs in good yields for 3-chlorocoumarins [26]. A rapid and effective synthetic route to obtain 3-phenylcoumarins from 3-chlorocoumarin, involving a Suzuki-cross-coupling reaction, using a catalytic complex Pd-salen and a series of different substituted boronic acids, has been described [27]. The Suzuki–Miyaura cross-coupling of 3-chlorinated-4-alkoxycoumarins with a range of aryl- and heteroarylboronic acids has been carried out in the presence of Pd(OAc)_2_ and 2-(dicyclohexylphosphino)-2,6-dimethoxybiphenyl (SPhos) in environmentally friendly conditions, and yields up to 99%. Sensitive functional groups, such as aldehydes and nitriles, were tolerated under the reaction conditions, and the protocol was scaled-up to a gram. The demethylation of the Suzuki–Miyaura products allowed obtaining 3-aryl-4-hydroxycoumarins. Chlorination of the coumarin framework was achieved by using trichloroisocyanuric acid (C_3_Cl_3_N_3_O_3_), which has several advantages over *N*-chlorosuccinimide, particularly regarding cost-effectiveness and toxicity [28].

### 3.2. Construction of the Coumarin Bicyclic System

**Figure 4:**
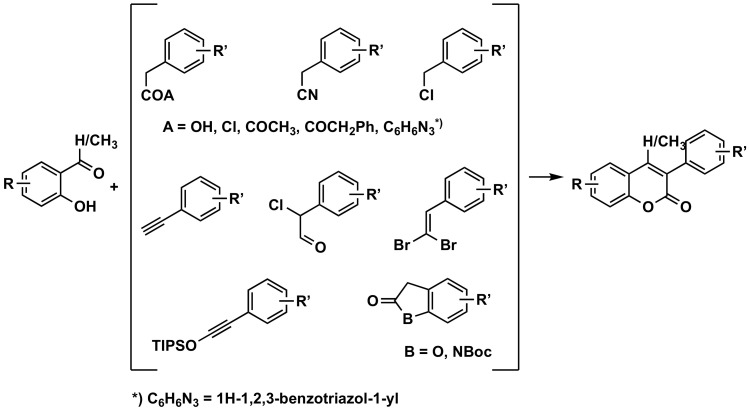
One of the synthetic routes to obtain 3-phenylcoumarins: construction of the coumarin from *o*-hydroxybenzenecarbonyls.

**Figure 5:**
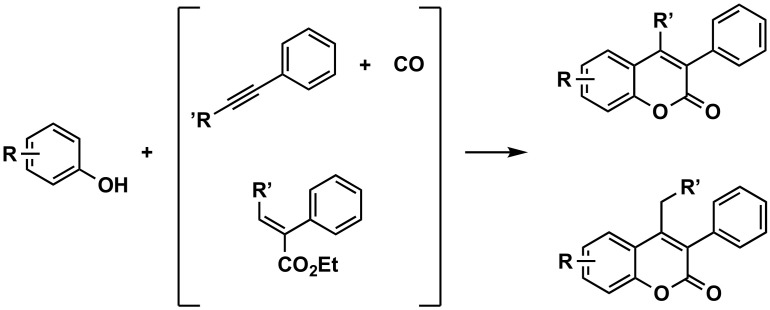
One of the synthetic routes to obtain 3-phenylcoumarins: construction of the coumarin from phenols.

**Figure 6:**
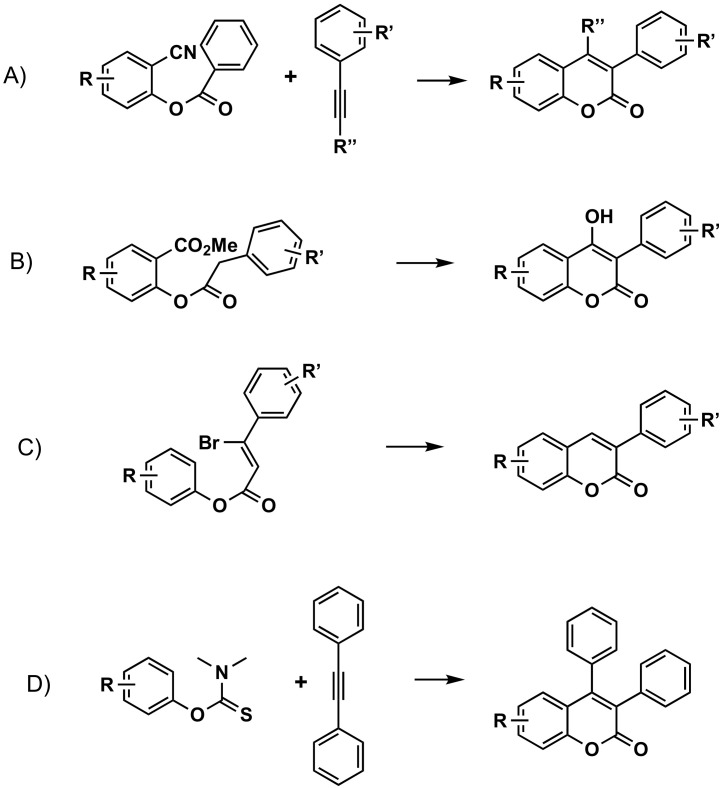
Four of the synthetic routes to obtain 3-phenylcoumarins: construction of the coumarin from phenolic esters and thiocarbamates. (**A**) Intermolecular cycloaddition reaction with the cleavage of two independent C–CN and C–CO bonds in an unusual mechanism. (**B**) Intramolecular Claisen condensation reaction of methyl 2-(2-arylacetoxy)benzoates. (**C**) Microwave irradiation of aryl-3-bromoacrylates. (**D**) Rhodium-catalyzed annulation of aryl thiocarbamates with internal alkynes, through C-H bond activation.

**Figure 7:**
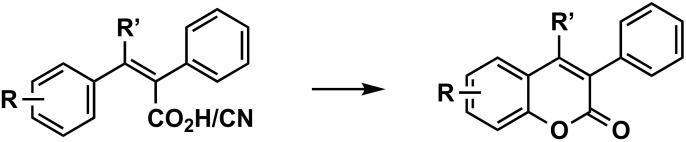
One of the synthetic routes to obtain 3-phenylcoumarins: construction of the coumarin from cinnamic acids.

**Figure 8:**
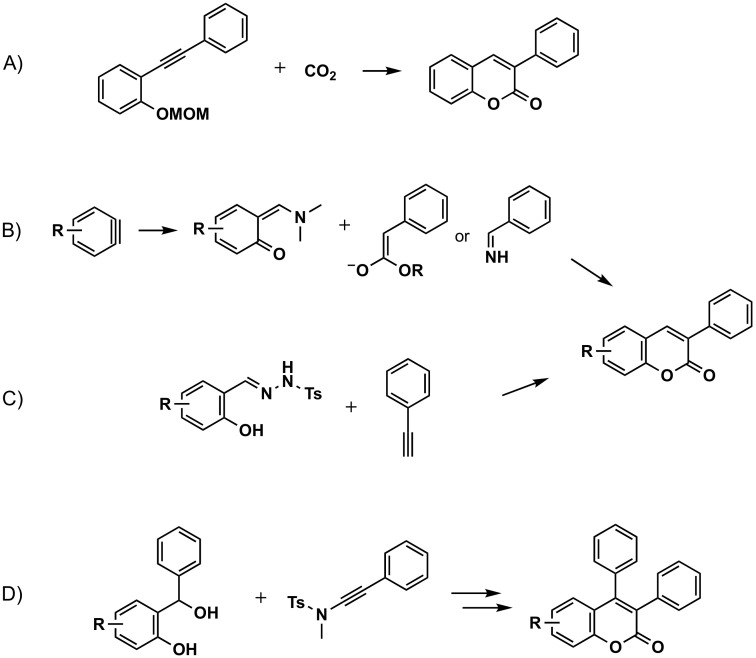
Four of the synthetic routes to obtain 3-phenylcoumarins: construction of the coumarin from other aromatic substrates. (**A**) One-pot synthesis of 3-substituted coumarins employing a phenyl alkyne with a MOM protected hydroxyl group as the ancillary substituent. (**B**) Direct access to coumarins through [4 + 2] cycloaddition between o-quinone methides, generated by a [2 + 2] cycloaddition from arynes. (**C**) Regioselective green synthesis of 3-arylcoumarins via visible-light-driven copper catalyzed aerobic oxidative cascade cyclization. (**D**) Synthesis of trans-3,4-diaryldihydrocoumarins in a regio- and diastereoselective reaction, via metal-free [4 + 2] annulation of ynamides with o-hydroxybenzyl alcohols, followed by oxidation with DDQ.

This strategy is the most commonly used to prepare 3-arylcoumarins and, of all the different options, the most explored occurs using the corresponding *o*-hydroxybenzenecarbonyls, an aldehyde (most frequent) or a ketone (Figure 4, Figure 5, Figure 6, Figure 7 and Figure 8).

#### 3.2.1. From *o*-hydroxybenzenecarbonyls

##### By Reaction with Phenylacetic Acids

Many one-pot and two-step esterification–cyclization methods to obtain 3-arylcoumarins are found in the literature. A wide variability of acids and also of *o*-hydroxybenzaldehydes or *o*-hydroxybenzoketones can be used, as well as a large number of acid-activating agents or coupling agents. 3-Arylcoumarins, with different substitution patterns, have been prepared in good yields via a direct synthetic route involving a classical Perkin reaction by condensation of appropriately substituted salicylaldehydes with phenylacetic acids, using *N*,*N’*-dicyclohexylcarbodiimide (DCC) as the dehydrating agent [29,30]. The activation of the acid can occur with thionyl chloride and intramolecular cyclization in an alkaline medium, in very good yields [31]. This acid activation can be performed using many other approaches, such as using cyanuric acid [32], always with good yields. Different types of coupling agents can also be used, such as propane phosphonic anhydride (T3P), a mild and low toxic coupling agent [33], or 1,4-diazabicyclo [2.2.2]octane (DABCO) [34]. Ph_3_P/I_2_–Et_3_N-mediated reactions [35] through microwave radiation heating have been described to be environmentally friendly, economic, easy to purify, less contaminant (less by-products formed) and high-yield reactions [36]. Recently, one-pot synthetic methodologies allowed obtaining diversely substituted 3-arylcoumarins using ionic liquids (IL), using 1-butyl-3-methylimidazolium hexafluorophosphate (BMIM-PF6) or 1-butyl-3-methylimidazolium tetrafluoroborate (BMIM-BF4) as solvent, and piperidine-appended imidazolium ionic liquid [PAIM][NTf_2_] as a task-specific basic-IL [37]. Instead of using phenylacetic acids, many of the described synthetic methodologies already started with activated acid derivatives, such as chlorides with 1,8-diazabicyclo[5.4.0]undec-7-ene (DBU) [38] or K_2_CO_3_, using ultrasound irradiation [39], anhydrides in a modification of the Perkin reaction using activated barium hydroxide/dimethylsulfoxide [Ba(OH)_2_/DMSO] medium under microwave irradiation [40] or using mixed hydrides, which allow obtaining the carboxyl group on the benzene ring at position 3 [41]. An ultrasound-assisted one-pot acylation/cyclization reaction between *N*-acylbenzotriazoles and 2-hydroxybenzaldehydes has also been developed, in quantitative yields, for 24 examples, in a parallel synthesis [42]. The substitution of the phenylacetic acid for the corresponding phenylacetonitrile, in the presence of Potassium *tert*-butoxide (*t*BuOK) in dimethylformamide (DMF), was also performed in quantitative yields for 29 examples [43].

##### By Reaction with Other Synthons

A straightforward procedure for the synthesis of several 3-arylcoumarins via palladium-catalyzed carbonylation of salicylic aldehydes, carbon monoxide and benzyl chlorides, using 1,3-bis(diphenylphosphino)propane (DPPP) as ligand and triethylamine (NEt_3_) as base, in dioxane, has been developed in good to excellent yields [44]. A highly efficient palladium-catalyzed carbonylative annulation of salicylaldehydes with benzyl chlorides, using *N*-formylsaccharin—a low cost, solid and easy to handle reactive—avoids the use of the highly toxic carbon monoxide (CO) gas [45]. A series of 34 3-arylcoumarins has been synthetized, in yields higher than 90%, via the tandem reaction of salicylaldehyde with aryl-substituted 1,1-dibromo-1-alkene [46]. The 3-arylcoumarin scaffold can also be efficiently obtained through an annulation reaction with terminal alkynes and salicylaldehydes by simply switching to a rhodium catalyst [47]. A series of 24 3-arylcoumarins has been obtained in good to excellent yields by the condensation reaction of 2-chloro-2-arylacetaldehyde with salicylaldehyde, catalyzed by *N*-heterocyclic carbene [48]. Another series of 50 3-arylcoumarins has been obtained in high yields (73–97%), mild conditions, short reaction times, simple work-up and broad scope of reactants by an organocatalytic cascade synthetic protocol mediated by DBU or tetramethylguanidine via condensation-ring opening-annulation between *N*-bocindolin-2-ones/benzofuran-2(3*H*)-ones and salicylaldehydes [49]. Finally, 4-hydroxy-7-methoxy-3-phenylcoumarin has been prepared in 59% yield, using 1-(2-hydroxy-4-methoxyphenyl)-2-phenylethanone in the presence of sodium in diethyl carbonate [50].

#### 3.2.2. From Phenols

The carbonylative annulation of simple phenols and alkynes leads to the synthesis of 3-arylcoumarins in moderate to good yields with excellent regioselectivity and functional-group tolerance by using iridium as a catalyst and copper as the promotor, at atmospheric pressure (Figure 5) [51]. The carbonylation has been also carried out for the synthesis of 3-aryl-4-(arylethynyl)coumarins from 2-iodoaryl 2-arylacetates and arylacetylenes via a Sonogashira coupling–intramolecular aldol cascade reaction, in the presence of bis(triphenylphosphine)palladium chloride [Pd(PPh_3_)_2_(Cl)_2_] as the catalyst [52]. 3-Arylcoumarins have been obtained from the condensation of electron-rich arenes (phenols or methoxyphenols) with allenes, in the presence of trifluoromethanesulfonic acid (TfOH), in good yield. The substitution at position 4 depends on the substituent pattern of the employed allenes. Available allenes have been employed as the three-carbon atom sources to construct the coumarin scaffold [53]. Finally, a recent and efficient approach for the synthesis of 3-arylcoumarins from salicylaldehydes and siloxy alkynes via a new [4 + 2] cyclization, using triflimide (HNTf_2_) as a catalyst, has been carried out [54].

#### 3.2.3. From Phenolic Esters

Nickel-catalyzed cycloaddition of *o*-arylcarboxybenzonitriles involves a reaction with alkynes, in the presence of Ni(0)/P(CH_2_Ph_3_)/MAD as a catalyst, to afford coumarins. This is an intermolecular cycloaddition reaction with the cleavage of two independent C–CN and C–CO bonds in an unusual mechanism (Figure 6A) [55]. 3-Arylcoumarins have also been readily prepared trough an intramolecular Claisen condensation reaction of methyl 2-(2-arylacetoxy)benzoates in the presence of cesium carbonate (Cs_2_CO_3_)-acetone, in excellent yields, and with easy workup procedures (Figure 6B) [56]. Aryl-3-bromoacrylates can be cyclized into 3-arylcoumarins by microwave irradiation in DMF, in the presence of K_2_CO_3_, up to a 72% yield. Twenty-two examples, from which some are 3-arylcoumarins, were described (Figure 6C) [57]. Finally, 3,4-diarylcoumarins have been synthesized through a rhodium-catalyzed annulation of aryl thiocarbamates with internal alkynes, through C–H bond activation (Figure 6D) [58].

#### 3.2.4. From Cinnamic Acids

A variety of functionalized coumarins, including 3-arylcoumarins, have been synthesized from substituted phenylacrylic acids (Figure 7) through a combination of phenyliodine diacetate and iodine (PIDA/I_2_) as the catalyst and irradiation-promoted oxidative C–O bond formation, in 41–92% yields [59,60]. Several trisubstituted acrylic acids within an aryl group at the position β have been efficiently obtained via one-pot reaction, a nickel-catalyzed arylcarboxylation of alkynes with arylmagnesium reagents and carbon dioxide (CO_2_, 1 atm) [60]. 3-Arylcoumarins have been also conveniently synthesized in excellent yields by a ferric chloride (FeCl_3_)-promoted tandem reaction using (*E*)-2,3-diphenylpropenoic acids or their methyl esters [61]. Finally, an acrylonitrile derivative has also been synthesized with pyridinium hydrochloride, in the presence of silica gel, by using microwave irradiation [62].

#### 3.2.5. From Other Aromatic Substrates

A one-pot synthesis of 3-substituted coumarins has been obtained employing a phenyl alkyne with a methoxymethyl (MOM) protected hydroxyl group as the ancillary substituent; although, in the case of 3-phenylcoumarin, the yield was described to be less than 10% (Figure 8A) [63]. Direct access to coumarins has been carried out through [4 + 2] cycloaddition between *o*-quinone methides, generated by a [2 + 2] cycloaddition from arynes, DMF and active methylene compounds (ester enolates or ketenimine anions from arylacetic acid esters or arylacetonitriles), with yields highly dependent on the substitutions in the aromatic ring (Figure 8B) [64]. Regioselective green synthesis of 3-arylcoumarins has been carried out via visible-light-driven copper catalyzed aerobic oxidative cascade cyclization of *N*-tosylhydrazones with terminal alkynes (Figure 8C) [65]. The synthesis of *trans*-3,4-diaryldihydrocoumarins has been carried out in a regio- and diastereoselective reaction, via metal-free [4 + 2] annulation of ynamides with *o*-hydroxybenzyl alcohols. Further oxidation with 2,3-dichloro-5,6-dicyano-1,4-benzoquinone (DDQ) has given the 3,4-diarylcoumarins in good yields (Figure 8D) [66].

### 3.3. Construction of the Benzene Ring at Position 3 of the Coumarin

**Figure 9:**
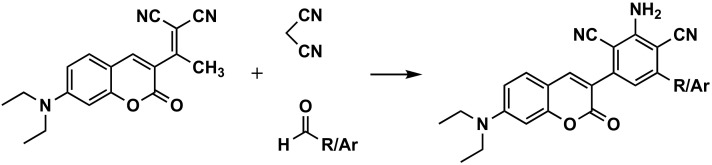
One of the synthetic routes to obtain 3-phenylcoumarins: construction of the benzene ring at position 3 of the coumarin.

As expected, this method is not the usually chosen for the preparation of 3-arylcoumarins and can only be an alternative when some special modification in that benzene ring at position 3 is of high interest (Figure 9). Therefore, only a simple and direct approach to the synthesis of coumarin-containing highly fluorescent asymmetric/symmetric 3,5-diaryl/heteroaryl-2,6-dicyanoaniline derivatives has been described in the past decade. The molecules have been synthesized in good yields via base-catalyzed three-component one-pot reaction of [2-(1-(7-(diethylamino)-3-coumarinyl)ethylidene]malononitrile with aliphatic, aromatic/heteroaromatic aldehydes and malononitrile, in solvent-free medium, catalyzed by piperidine, under a microwave irradiation method (Figure 9) [67].

## 4. Modifications of the 3-Arylcoumarin Scaffold

**Figure 10:**
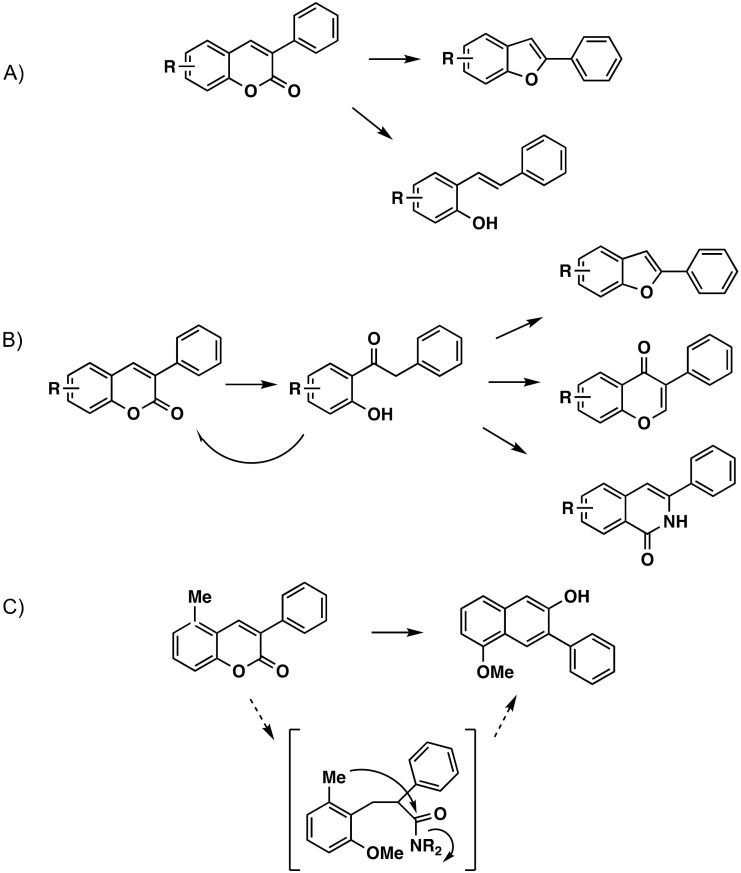
Three different reactions for the synthesis of new molecules with modifications of the 3-arylcoumarin scaffold. (**A**) Synthesis of a variety of 2-arylbenzofurans from 3-arylcoumarins, through a copper-catalyzed decarboxylative intramolecular CO coupling one-pot reaction. Synthesis of *o*-hydroxystilbenes from 3-arylcoumarins via hydrolysis/decarboxylation cascade reaction in the presence of KOH. (**B**) Simple, green and efficient approach to obtain 2-hydroxydeoxybenzoins, from the microwave-assisted alkali degradation of 3-aryl-4-hydroxycoumarins in water. (**C**) Synthesis of aryl-2-naphthols via a general and efficient one-pot methodology using highly substituted 2-naphthols, exploring the dual nature of lithium bases.

Different reactions are found which are related to this scaffold; however, in this section, only those for which their modification has been carried out are shown. A variety of 2-arylbenzofurans have been prepared from 3-arylcoumarins, with yields from 26% to 84%, through a copper-catalyzed decarboxylative intramolecular CO coupling one-pot reaction [68] (Figure 10A). A series of 3-arylcoumarins could readily undergo a hydrolysis/decarboxylation cascade reaction in the presence of potassium hydroxide (KOH), in ethylene glycol, to afford the desired *o*-hydroxystilbenes in moderate to high yields (Figure 10A) [69]. Through a simple, green and efficient approach, the synthesis of 2-hydroxydeoxybenzoins, from the microwave-assisted alkali degradation of 3-aryl-4-hydroxycoumarins in water, has been reported. A wide variety of cyclization reactions can occur from this intermediate (Figure 10B) [56]. A general and efficient one-pot methodology using highly substituted 2-naphthols has been performed, exploring the dual nature of lithium bases, consisting of consecutive ring opening of available coumarins with either lithium diethylamide (LiNEt_2_) or lithium diisopropylamide (LiNiPr_2_) into Z-cinnamamides. This generates a directing group in situ and allows, by conformational freedom, a lateral directed remote metalation for ring closure to give the aryl-2-naphthols in good to excellent yields (Figure 10C) [70].

## 5. Pharmacological Interest of 3-Arylcoumarins

Many scientific references have appeared regarding the pharmacological interest of 3-arylcoumarins during the past decade. In this review, all the available references have been collected and analyzed. The most relevant are included in this overview, but only the most attractive in each area are highlighted. Because there are a large number of different properties attributed to 3-arylcoumarins, they have been grouped into three large groups, divided in different subgroups. Occasionally, we come across publications that could be included in more than one group. For those, the inclusion criterion has been based on a greater affinity towards one of the groups.

### 5.1. Neuroprotective Agents

This section includes the most relevant bibliographic references that cover studies on neurodegenerative disorders, such as Alzheimer’s or Parkinson’s diseases, but also oxidation, inflammation, cardiovascular diseases and some related enzyme inhibitors, such as monoamine oxidases (MAO) or acetyl- and butyrylcholinesterase (AChE and BuChE), related to the subject.

#### 5.1.1. Alzheimer’s Disease and AChE

**Figure 11:**
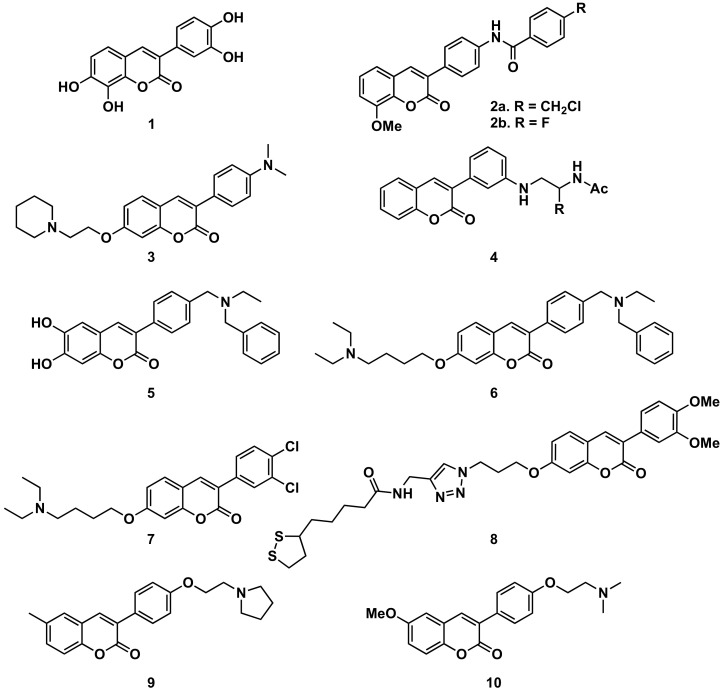
3-Phenylcoumarins in Alzheimer’s disease: AChE inhibitors.

Several series of simple 3-phenylcoumarins have been studied to prevent and treat Alzheimer’s disease and its complications, showing high activity toward AChE and MAO, together with antioxidant activity (Figure 11). Among a studied series of polyhydroxy 3-phenylcoumarins, 3-(3′,4′-dihydroxyphenyl)-7,8-dihydroxycoumarin (**1**) stands out with IC_50_ inhibition values 3 μM and 27 μM against AChE and MAO-B, respectively [71]. This research group also studied a series of benzamide derivatives at the position 4′, resulting in the compound **2**, the best of the series, with an AChE inhibition IC_50_ of 0.09 μM. The authors claim the possibility of partially modulating the AChE/BuChE selectivity by modifying the substitution of the chloromethyl group at *para* position of the benzamide group by a fluorine atom [72], showing another example of the interest of introducing a benzamide group on the coumarin scaffold for this activity [73]. The aniline fragment at position 3 of the coumarin has also been explored in a series of compounds with a 7-aminoalkoxy chain, resulting in 3-(4′-(dimethylamino)phenyl)-7-(2-(piperidin-1-yl)ethoxy)coumarin (**3**), the most interesting compound of those studied, showing an excellent AChE inhibition potency (IC_50_ = 20 nM) and selectivity (IC_50_ BuChE/AChE = 354), quite similar to the reference drug (IC_50_ donepezil = 6 nM; IC_50_ BuChE/AChE = 365) [74]. Also, from 3-(3′-aminophenyl)coumarin (*K*_i_ = 146 μM), a non-peptidic drug-like β-secretase 1 (BACE-1) inhibitor, the hit fragment containing the *N*-acylated ethane-1,2-diamine motif has been identified as a directing probe to pick inhibitory fragments for the S1 pocket of the aspartic protease BACE-1, leading to the most interesting compound (compound **4**, with a biphenylmethyl residue) with a *K*_i_ of 3.7 μM [75]. Separating the amine from the benzene ring at position 3 of the 3-phenylcoumarins by a methylene group proved to be an interesting strategy to obtain more active compounds. When this amine is a bulky amine as a *N*,*N*-dibenzyl(*N*-ethyl)amine fragment, several compounds of a studied series proved to act on three relevant targets in Alzheimer’s disease: σ-1 receptor (σ1R), BACE1 and AChE. These also show potent neurogenic properties, good antioxidant capacity and favorable central nervous system (CNS) permeability. Compound **5** must be highlighted within the series [76]. Compounds with the same substitution at position 4′, and a 7-aminoalkoxy chain, formed a series of products presenting multitarget interest for the treatment of the middle stage of Alzheimer’s disease. A non-neurotoxic dual AChE/BuChE inhibitor, compound **6**, which is also a nanomolar human AChE inhibitor, turned out to be a significant inhibitor of Aβ42 self-aggregation activity, being also a promising neuroprotective agent [77]. A series of compounds with 7-aminoalkoxy-3-phenylcoumarins, presenting simple substitutions on the phenyl at position 3, were studied, identifying compound **7** as the most potent compound against AChE (IC_50_ = 0.27 μM). Kinetic and molecular modelling studies proved that compound **7** works in a mixed-type approach, and interacts concomitantly with the catalytic active site (CAS) and the peripheral anionic site (PAS) of AChE. In addition, compound **7** blocks β-amyloid (Aβ) self-aggregation with a ratio of 44% at 100 μM, and significantly protects rat pheochromocytoma (PC12) cells from hydrogen peroxide (H_2_O_2_)-damage in a dose-dependent way [78]. The 7-substitution of 3-phenylcoumarins has also been used as a building block for a novel series of coumarin-lipoic acid conjugates, resulting in compound **8**, the most potent AChE inhibitor, showing a good inhibitory effect on Aβ-aggregation and intracellular ROS formation, as well as the ability of selective bio-metal chelation and neuroprotection against H_2_O_2_- and Aβ1-42-induced cytotoxicity [79,80]. Interesting to note is the comparison between two compounds prepared in a study on Alzheimer’s disease, being 6-substituted 3-arylcoumarins. Compounds **9** and **10**, with the substituents in the same positions, and small structural differences between them, do not offer great differences in their activities and selectivity against cholinesterases or towards the inhibition of self-induced Aβ42-aggregation; however, they do offer important differences in selectivity against human MAO-A and MAO-B which are worthy of further study [81].

#### 5.1.2. Parkinson’s Disease and MAO-B

**Figure 12:**
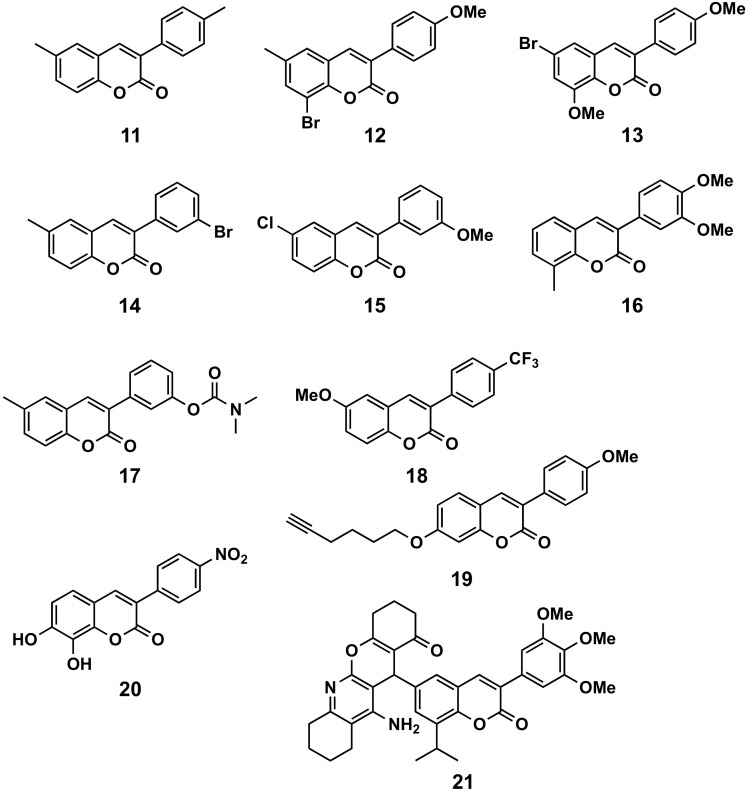
3-Phenylcoumarins in Parkinson’s disease: MAO-B inhibitors.

Several articles have been published regarding the study of the inhibitory capacity and selectivity of different 3-phenylcoumarins on human MAOs, and their interest as possible anti-Parkinson’s compounds (Figure 12). In many cases, simple backbone substitutions led to excellent results against MAO-B. We may say that this is the most promising pharmacological application of simple 3-phenylcoumarin derivatives. Compounds with only methyl groups in different positions of the 3-phenylcoumarins scaffold led to very interesting MAO-B inhibitors, i.e., the 6-methyl-3-(*p*-tolyl)coumarin (**11**), with an IC_50_ = 308 pM [82] and the 8-methyl-3-(*p*-tolyl)coumarin, with an IC_50_ = 4.51 nM [83]. Additionally, most of these molecules are selective and reversible MAO-B inhibitors. Also, compounds with methyl groups, and other additional substituents, have shown interesting properties. For the analysis of compounds with a bromine atom on the benzene ring of the coumarin scaffold, different compounds with other methyl groups have been synthesized, i.e., the 8-bromo-6-methyl-3-(4′-methoxyphenyl)coumarin (**12**), with an IC_50_ of 3.23 nM. This compound presents an IC_50_ that has half the value of selegiline (IC_50_ = 19.60 nM) and more than a 31,000-fold MAO-B selectivity index (SI) against MAO-A [84]. Also, some molecules presenting other methoxy groups on their structures, besides the bromine atom, i.e., the 6-bromo-8-methoxy-3-(4′-methoxyphenyl)coumarin (**13**), with an IC_50_ of 1.35 nM, are even better than 8-bromo-6-methyl-3-(4′-methoxyphenyl)coumarin and selegiline [85]. Some compounds with the bromine atom on the benzene ring at the 3′ position, i.e., the 3-(3′-bromophenyl)-6-methylcoumarin (**14**) (IC_50_ = 134 pM), proved to be the most active compounds within the studied series, being 140-fold more active than selegiline, and showing the highest SI toward human MAO-B [86]. In a study on the effect of the substitution at position 6 of the 4-hydroxy-3-phenylcoumarins, it is shown that the presence of a chlorine atom improves the inhibitory activity, as well as the selectivity, against MAO-B, compared to non-substituted or the substituted by a methyl group [87]. These compounds are even more active when position 4 is non-substituted, thus obtaining, i.e., the 6-chloro-3-(3′-methoxyphenyl)coumarin (**15**), with an IC_50_ of 1 nM [88]. PEGylated [poly(lactide-co-glycolide)] PLGA nanoparticles have been described as smart delivery carriers, as well as having been used to try to solve the suboptimal aqueous solubility of 3-arylcoumarins [89]. The study has been carried out with the 3-(3′,4′-dimethoxyphenyl)-8-methylcoumarin (**16**), a potent, reversible and selective MAO-B inhibitor (IC_50_ = 28.89 nM). The encapsulation efficacy was around 50%, leading to a final concentration of 807 μM in the nanoformulation, which corresponds to a therapeutic concentration 27828-fold higher than the IC_50_. This compound proved to show some cytotoxic effects only at 50 μM, after 48 and 72 h of exposure to human colorectal adenocarcinoma (Caco-2), human neuroblastoma (SH-SY5Y) and human endothelial (hCMEC/D3) cell lines. Remarkably, no cytotoxic effects have been observed for the compound formulated in the nanoparticles. Furthermore, the data obtained showed that this coumarin is a P-glycoprotein substrate, displaying a lower uptake profile in intestinal and brain endothelial cells [89]. Therefore, the encapsulation proposed by the authors seems to be an excellent strategy to avoid the partial toxicity of the compound, and the cell expulsion via P-glycoprotein systems. 3-(3′-Dimethylcarbamatephenyl)-6-methylcoumarin (**17**), with a carbamate group at position 3′, has been identified as the most potent of a studied series of 6-methyl derivatives, with an IC_50_ of 60 nM, and SI higher than 1664-fold [90]. 6-Methoxy-3-(4′-(trifluoromethyl)phenyl)coumarin (**18**, IC_50_ = 56 nM) has been identified as the most potent of a wide series of derivatives with fluorine atoms on the phenyl ring at position 3 [91]. In a study of alkynylcoumarinyl ethers, it has been found that the most convenient position for a lateral chain is position 7 (from positions 6, 7 or 8), being the best chain a hex-5-ynyloxy. On this structure, a methoxy group at position 3′ has been characterized as a dual-acting inhibitor, with IC_50_ values of less than 10 nM towards MAO-A and MAO-B. 3-(4′-Methoxyphenyl)coumarin derivative **19** proved to combine a strong MAO-B inhibitory activity (IC_50_ = 3.0 nM), with a high SI for MAO-B over MAO-A (>3400-fold) [92]. A 4-nitro derivative **20** has been identified as the most active of a small series studied with substitutions containing oxygen atoms; however, it turned out to be non-selective (MAO-A IC_50_ = 6.5 μM and MAO-B IC_50_ = 3.8 μM [93]. In a comparison between the substitution by nitro or amino groups in the *meta* or *para* positions of the phenyl at position 3, the substitution by amino groups proved to be more interesting [94]. A series of multifunctional directed 3-arylcoumarin-tetracyclic tacrine derivatives has been evaluated for the treatment of Parkinson’s disease. A number of derivatives (being compound **21** the most active) demonstrated a significant reduction in the aggregation of human α-synuclein, and also a significant increase in dopamine [95]. Finally, in some cases, 3D-QSAR (quantitative structure-activity relationship) and/or molecular modelling methods have been used to try to enhance the probability of finding new interesting inhibitors [52,96,97,98]. In some cases, comparison between scaffolds, such as 3-phenylcoumarins and 2-arylbenzofurans, has also been carried out through docking calculations [99].

#### 5.1.3. Inflammation

**Figure 13:**
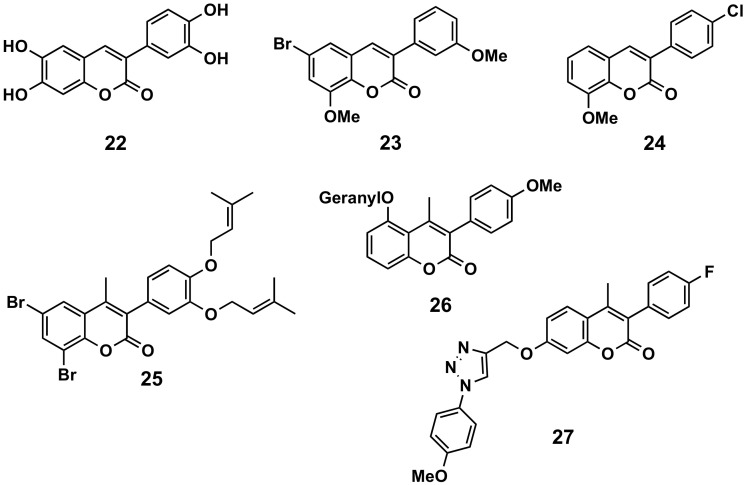
3-Phenylcoumarins in inflammation.

In a comparative study, 6,7-disubstituted compounds proved to down-modulate the Fc-gamma (Fcγ) receptor-mediated neutrophil oxidative metabolization more efficiently than compounds disubstituted at positions 5 and 7, and hydroxylated compounds down-modulated this function more strongly than their acetylated counterparts. The most interesting compound turned out to be compound **22** and, even better, their 3′,4′-methylenedioxyphenyl derivative, which may be a prototype for the development of novel immunomodulating drugs to treat immune complex-mediated inflammatory diseases (Figure 13) [100]. A series of 34 3-phenylcoumarins has been evaluated in lipopolysaccharide-activated mouse macrophage RAW264.7 cells. 6-Bromo-8-methoxy-3-(3′-methoxyphenyl)coumarin (**23**), a dimethoxybromine derivative, exhibited nitric oxide production inhibitory activity, with an IC_50_ of 6.9 μM [101]. Some simple derivatives such as the 3-(4-chlorophenyl)-8-methoxycoumarin (**24**) showed interesting soybean lipoxygenase inhibitory activities due to the importance they may have in anti-inflammatory processes [34]. Prenyloxycoumarin **25** displayed the best combined inhibition of lipid peroxidation (100%) and soybean lipoxygenase (IC_50_ = 37 μM) amongst the studied series of coumarins and thiocoumarins [102]. The geranyloxy-derived compound **26** also exhibited good soybean lipoxygenase inhibition (IC_50_ = 10 μM) [103]. Finally, searching for anti-inflammatory activities, but also antimicrobial and antioxidant properties, a series of 3-phenylcoumarins with a 1,2,3-triazole 1,4-disubstituted residue attached by an ether to the position 7 of the scaffold, has been prepared. Compound **27** proved to be the most interesting pharmacological profile, with an IC_50_ of 15.78 μM (by the egg-albumin), slightly higher than diclofenac (IC_50_ = 17.52 μM) [104].

#### 5.1.4. Oxidation

**Figure 14:**
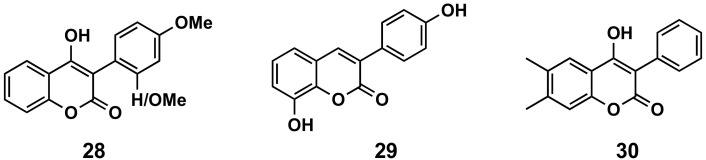
3-Phenylcoumarins in oxidation.

2′- or 4′-Methoxy derivatives of 4-hydroxy-3-phenylcoumarins (compound **28**, Figure 14) have shown higher antioxidant capacity than 4-hydroxycoumarin, measuring their capacity to scavenge two different radicals: 2,2-diphenyl-1-picrylhydrazyl (DPPH) and 2,2′-azinobis-3-ethylbenzothiazoline-6-sulfonic acid (ABTS). These molecules also display protecting effects towards the β-carotene-linoleic acid co-oxidation enzymically induced by lipoxygenase [105]. From a series of hydroxylated 3-phenylcoumarin derivatives [106,107], 8-hydroxy-3-(4′-hydroxyphenyl)coumarin (**29**, Figure 14) has been the most interesting molecule, showing an oxygen radical absorbance capacity fluorescein (ORAC-FL) of 13.5, capacity of scavenging hydroxyl radicals of 100%, capacity of scavenging DPPH radicals of 65.9% and capacity of scavenging superoxide radicals of 71.5%, as well as being a potential candidate for preventing or minimizing the free radicals’ overproduction in oxidative-stress related diseases [106]. Simple 3-phenylcoumarins (in many cases associated with other cycles) have been identified as inhibitors of the function of nicotinamide adenine dinucleotide phosphate (NADPH) and quinone reductase (NQO1), with multiple cellular functions as detoxifying enzymes, as well as chaperone proteins. These compounds proved to be even more potent and less toxic than dicoumarol, and more efficient for inhibiting the toxicity of chemotherapeutic drug EO9 [108,109]. 4-Hydroxy-6,7-dimethyl-3-phenylcoumarin (**30**, Figure 14) stands out with an IC_50_ of 660 nM in the presence of bovine serum albumin (BSA) [109]. Finally, QSAR predictive models for antioxidant activity of new coumarin derivatives have also been described as an interesting tool in drug discovery [110].

#### 5.1.5. Cardiovascular Diseases

**Figure 15:**
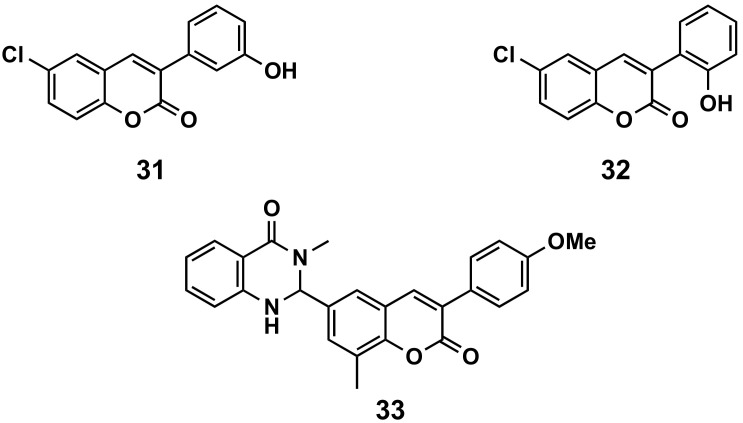
3-Phenylcoumarins in cardiovascular diseases.

A series of 6-halo-3-hydroxyphenylcoumarins has been evaluated for their vasorelaxation activity in intact rat aorta rings pre-contracted with phenylephrine, as well as for their inhibitory effects on platelet aggregation induced by thrombin in washed human platelets (Figure 15). These compounds proved to relax the vascular smooth muscle in a concentration-dependent manner. Compound **31** presents an IC_50_ of 36.6 μM against a concretion induced by phenylephrine. Some of the compounds showed a platelet antiaggregatory activity that was up to 30 times higher than that shown by *trans*-resveratrol, used as control, i.e., compound **32** (IC_50_ = 6.41 μM) [111]. The niacin receptor 1 (GPR109a) is a receptor that inhibits lipolytic and atherogenic activity and induces vasodilatation. A series of coumarin-dihydroquinazolinone conjugates has been evaluated for its agonist potential, displaying, in compound **33**, robust agonist action to GPR109a with an EC_50_ < 11 nM. Further, the efficacy of the active compound has been corroborated by in vivo assays, showing the animals reduced body weight in a diet-induced obese mice model. Compound **33** proved to reduce leptin in blood plasma and total serum cholesterol [112].

### 5.2. Chemotherapy

This section includes the most relevant bibliographic references that describe studies on bacteria, fungi, mycobacteria, viruses, parasites and cancer, as well as all the information somehow related to these topics.

#### 5.2.1. Antimicrobial Agents

**Figure 16:**
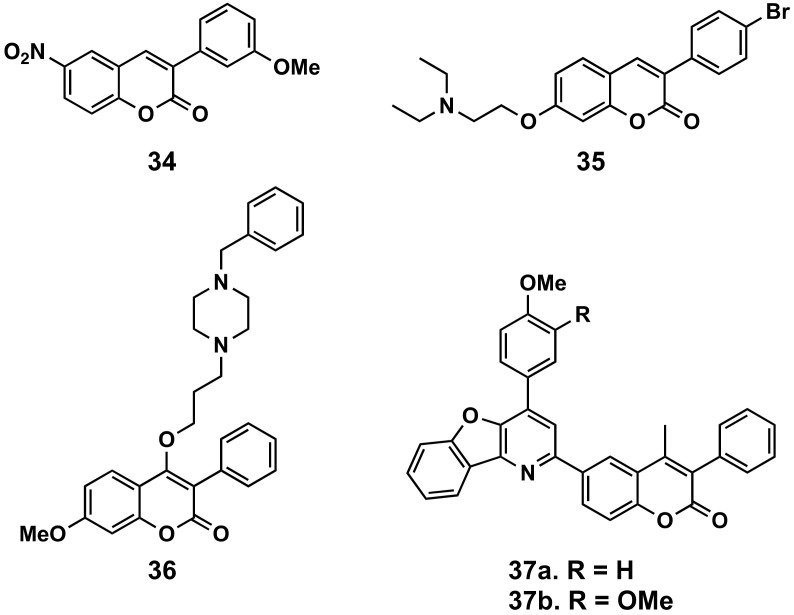
3-Phenylcoumarins as antimicrobial agents.

Traditionally, and since the development of novobiocin, an aminocoumarin antibiotic produced by the actinomycete *Streptomyces niveus*, coumarins have been studied as potential antimicrobial agents. Within this family of compounds, 3-phenylcoumarins have aroused some interest in the last decade (Figure 16). Three novel coumarin compounds, one of them a 3-phenyl derivative bearing an ethynyl group at position 4′, have been isolated from a methanol extract of the red ants of ChangBai Mountain, called *Tetramorium* sp. (Table 1). These new compounds exhibit significant inhibitory activity against Gram-positive bacteria *Bacillus subtilis*, presenting minimum inhibitory concentration (MIC) values of 25 μg/mL [2]. Some other compounds from a selected series of nitro/amino derivatives (compounds **34**) show antibacterial activity against another Gram-positive bacteria, *Staphylococcus aureus*, comparable to the described standards (oxolinic acid and ampicillin) [113]. Another series of 3-phenylcoumarins presented MICs of 150 mM against *S. aureus*, displaying activities in nanomolar concentrations against Gram-positive (*Staphylococcus aureus*, methicillin-resistant *S. aureus* (MRSA) and *Enterococcus faecium*), when coordinated to the fac-[Re(CO)_3_]^+^. These conjugates proved to be non-toxic in vivo, in zebrafish models, even at higher doses compared to the MIC values. These compounds do not affect the bacterial cell membrane potential. However, some of the most potent complexes strongly interact with DNA, indicating that this is a possible mechanism of action [114]. A series of compounds presenting aminoalkoxy groups at position 7 of the 3-phenylcoumarin showed significant activities against selective strains, i.e., *Trichophyton mentagrophytes*, with compound **35** being the best within the series, presenting better activity than fluconazole (MIC = 1.56 μg/mL) [115]. Compounds with a (4-benzylpiperazin-1-yl)propoxy substitution at position 4 of 3-phenylcoumarins (compound **36**) exhibited significant antibacterial and antifungal activity as that of standard streptomycin and itrazole, respectively [116]. Finally, compounds with benzofuro[3,2-b]pyridine core at the position 6 of the scaffold have been evaluated against *S. aureus*, *B. subtilis*, *Escherichia coli*, *Salmonella typhi*, *Candida albicans*, *Aspergillus niger* and *Mycobacterium tuberculosis* H37Rv, and exhibited promising activities, with compound **37** being the best compound within the series [117].

#### 5.2.2. Antiviral Agents

**Figure 17:**
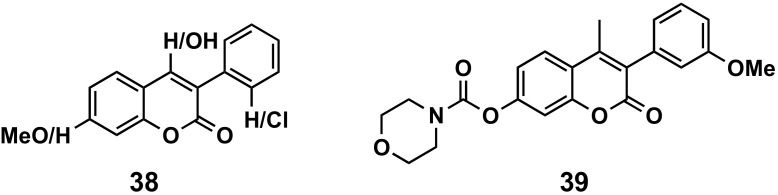
3-Phenylcoumarins as antiviral agents.

Only one study focused on experimental data on human immunodeficiency virus (HIV) has been found (Figure 17). Several 4-hydroxy-3-phenylcoumarins (compounds **38**), with small substitutions on the scaffold, inhibit HIV replication with IC_50_ values < 25 μM [118]. In addition, by screening chemical libraries in the RIKEN Natural Products Depository, coumarin-based compounds, such as 3-phenylcoumarins, have been identified as inhibitors of the cell cycle arrest activity of viral protein R Vpr, which has been shown to have multiple roles in HIV-1 pathogenesis. The binding of the carbamate derivative **39** may be produced by direct binding on the hydrophobic region, close to the Glu-25 and Gln-65 residues of the protein [119].

#### 5.2.3. Antiparasitic Agents

**Figure 18:**
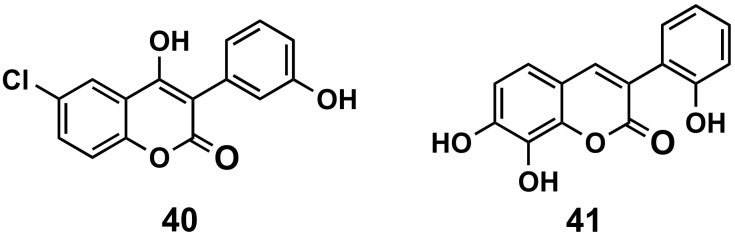
3-Phenylcoumarins as antiparasitic agents.

Our research group published two manuscripts based on the interest of hydroxylated 3-phenylcoumarins against *Trypanosoma cruzi*, the parasite responsible for Chagas disease (Figure 18). In addition, the antioxidant activities of the compounds have also been addressed. The 4-hydroxy derivatives showed only moderate results, although very good antioxidant capacity, such as the 6-chloro-4-hydroxy-3-(3′-hydroxyphenyl)coumarin (**40**), ORAC-FL = 7.7) [120]. The most interesting profile of trypanocide has been found for 7,8-dihydroxy-3-(2′-hydroxyphenyl)coumarin (**41**), presenting a moderate scavenging ability for peroxyl radicals (ORAC-FL = 2.23) and a high degree of selectivity towards the epimastigote stage of the parasite *T. cruzi* (IC_50_ = 1.31 μM), higher than Nifurtimox (drug currently used for the treatment of Chagas disease) [121].

#### 5.2.4. Anticancer Agents

**Figure 19:**
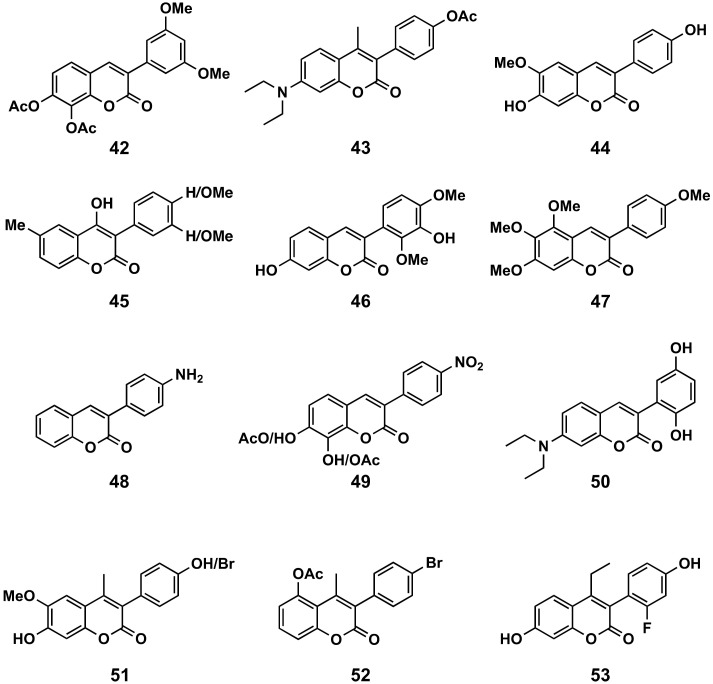
3-Phenylcoumarins as anticancer agents: simple coumarins, without the incorporation of new cycles to the scaffold.

**Figure 20:**
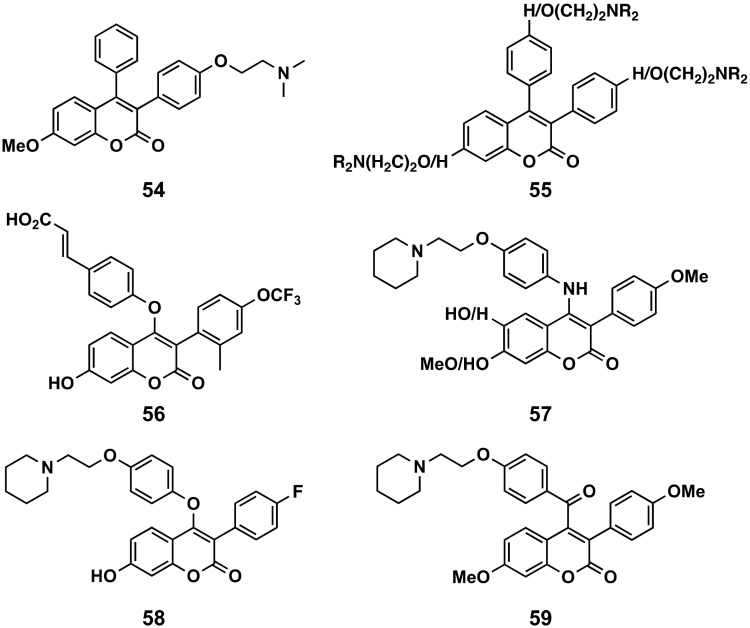
3-Phenylcoumarins as anticancer agents: coumarins with a benzene ring bound directly or not to position 4 of the 3-phenylcoumarin.

**Figure 21:**
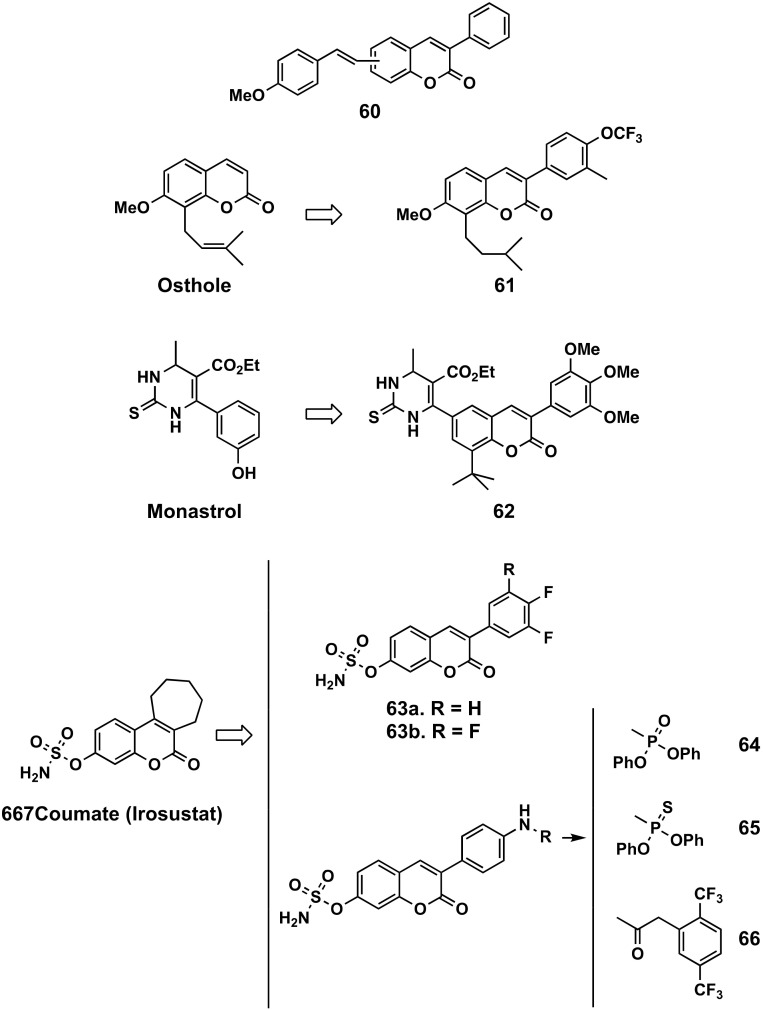
3-Phenylcoumarins as anticancer agents: coumarin hybrids or inspired by other already known compounds.

The most interesting compounds of the studied family displaying anticancer activity are represented and grouped in three different figures: (A) simple 3-phenylcoumarins, without the incorporation of new cycles to the scaffold (Figure 19), (B) 3-phenylcoumarins with a benzene ring bound directly or not to the position 4 of the 3-phenylcoumarin (Figure 20) and (C) 3-phenylcoumarin hybrids or those inspired by other already known compounds (Figure 21).

##### 3-Phenylcoumarins with Simple Substitutions

Simple coumarins are still the most studied compounds from this family. They are usually found in natural extracts, they are chemically simple structures and are therefore easy to synthetize and their simplicity allows multiple spots to include different and more complex substitution patterns. In this context, it is interesting to highlight the remarkable effect against KERATIN-forming (KB) tumor cells of the compound **42** (IC_50_ = 5.18 μM) [122]. In addition, coumarin **43** also presents an interesting effect on the growth of adenocarcinomic human alveolar basal epithelial cells (A549) (LD_50_ = 48.1 μM) and liver cancer cells (CRL 1548) (LD_50_ of 45.1 μM) [123]. In this same cell line, as well as human promyelocytic leukemia cells (HL-60), compound **44** has been the most interesting of the series with LD_50_ of 7.5 and 5.2 μM, respectively [124]. From a series of 4-hydroxy-3-phenylcoumarins studied in breast adenocarcinoma cells (MCF-7) and HL-60, compound **45** has been the best candidate [125]. Compound **46** proved to be an interesting candidate against A549, a pancreatic cancer cell line (Panc-28), and human colorectal carcinoma cells (HCT-116), with GI_50_ values of 7.39, 25 and 19.17 μM, respectively [3]. Novel polyalkoxy-3-(4′-methoxyphenyl)coumarin analogues, i.e., compound **47** and natural antimitotic compounds, have been synthesized from plant allylpolyalkoxybenzenes and tested in vivo in the phenotypic sea urchin embryo assay for antiproliferative antitubulin activity [126]. The described 4′-amino-derivative **48** induces Hes 1 downregulation and displays antitumor effects against HCT-116 cells [127]. A compound with a nitro group at 4′ and a hydroxy group at C-7 and C-8 (compound **49**) displayed high activity (IC_50_ = 17.65 μM) in human liver cells (HepG2) through a ROS-independent cell death mechanism [128]. Previously, the same research group had shown that the diacetylated derivative at 7 and 8 (diacetylated compound **49**) turned out to be the most interesting of a small series studied with the nitro group at position 4′, showing in vitro cytotoxic effect on A549, breast (MDA-MB-231) and prostate (PC3) cancer cell lines [129]. Very interesting to note is the determination that PBQC, an oxidized form of 7-diethylamino-3-(2′,5′-dihydroxyphenyl)coumarin (**50**), is a new nuclear factor-erythroid 2-related factor 2 (Nrf2) activator, which is a key factor involved in the regulation of cancer cell growth by the expression of pro-apoptosis genes [130]. On a series of 7-hydroxy-6-methoxy-4-methyl-3-phenylcoumarin derivatives, which have been considered non-steroidal analogues of 2-methoxyestradiol, the effects of specific substituents at position 4′ on the anti-angiogenesis activities have been investigated with human umbilical vein endothelial cells (HUVECs) proliferation assays. The most interesting compounds were the 4′-hydroxy and the 4′-bromo (compounds **51**), with IC_50_s of 61.0 and 76.7 μM, respectively [131]. The 4′-bromo substitution proved to be of remarkable interest against cervical cancer cells (HeLa), showing compound **52** with an IC_50_ of 1.8 μM [103]. Compound **53** exhibited an excellent ERα antagonistic activity (IC_50_ = 12 nM) and antiproliferative potency against MCF-7 cells, similar to tamoxifen [132]. The sphenostylisin A (Figure 2) has been found to be a very potent NF-κB inhibitor (IC_50_ = 6 nM) with promising interest in cancer and inflammation [4].

##### 3-Phenylcoumarins with Rings at Position 4

Novel triphenylethylene-coumarin hybrids, containing different amino side chains, have been studied. The effect against A549 cells of the compound **54** (IC_50_ = 5.0 μM) is remarkable [133,134]. Some derivatives which possess two amino side chains (except morpholinyl) at positions 4′ of one or both of the extra benzene rings, and/or at position 7 of coumarin scaffold (compounds **55**), proved to be more potent than tamoxifen against ER(+) MCF-7 and ER(−) epithelial human breast cancer (MDA-MB-231) cells, with IC_50_ < 10 μM, and showed a broad-spectrum and good anti-proliferative activities, with an intercalative mode of binding against five tumor cells and low cytotoxicity in osteoblast [135,136]. Both the DNA binding properties and the anti-proliferative activities would be enhanced by dimerization of the monomeric hybrid with one amino side chain between the phenyl groups at position 3 of coumarin, being significantly affected by the length of the linker [137,138]. More recently, it has been shown that the coumarin with a piperidineethoxy chain in *para* position of the 4′-phenyl, and the same chain at position 7, exerts its anti-cancer effects by inhibiting endothelial cell proliferation, migration and by suppressing tube formation and angiogenesis induced by breast cancer cells, both in vitro and in vivo [139]. Compound **56**, with a cinnamic acid linked to an ether function to the position 4, and a trifluormethyl group at position 4′ and powerful ER down-regulation (IC_50_ = 3 nM), is the result of the pharmacomodulation of other 7-hydroxycoumarin precursors with previously reported antitumor effects against chemically induced mammary tumors such as selective estrogenic receptor down-regulators [26]. A series of 18 4-anilino derivatives has been designed to obtain novel selective ERα modulators, and their antiproliferative activity has been evaluated against MCF-7 and Ishikawa cell lines, showing, for most of the derivatives, higher activity than tamoxifen, with the best being compound **57**, with an IC_50_ value of 4.52 μM [140]. In a 4-anilino/aryloxy comparison, compound **58** has been the most interesting molecule of the series as a dual ERα selective antagonist modulator/VEGFR-2 inhibitor [141]. The ERα binding affinity (IC_50_ = 2.19 mM) occurs via suppressing the expression of progesterone receptor (PgR) mRNA in MCF-7 cells, and, in the same cells, may inhibit the activation of VEGFR-2 and subsequent signaling transduction of the Raf-1/MAPK/ERK pathway [141]. Compounds with similar substitutions at position 4, but with variability at position 3 of the coumarin scaffold, have been evaluated in vitro against MCF-7, HepG2, HCT-116 and human pancreatic carcinoma (Panc-1) cell lines by the same authors, displaying antiproliferative activities comparable to fluouracil. However, compounds substituted by the 3-trifluoroacetyl group at position 3 of the coumarin scaffold proved to be the best candidates [142]. The series of compounds with aroyl substitutions, both at positions 4 and 6 of the 3-arylcoumarin scaffold, proved to be interesting compounds. Amongst these, compounds with substitutions at position 4, such as compound **59**, exhibit the most remarkable antiproliferative potential against both ER+ and ER− breast cancer cell lines, promoting alkaline phosphatase activity as well as enhancing osteoblast mineralization in vitro [143].

##### Hybrid 3-Phenylcoumarins

Thirteen coumarin-resveratrol hybrids (6 or 7-styryl-3-phenylcoumarins, compound **60**) have been designed, synthesized and evaluated for their antitumor activities against MCF-7, human colorectal carcinoma cells (HCT-28) and human immortalized myelogenous leukemia (K562) tumor cell lines. The most active compounds showed different degrees of cell growth inhibition (IC_50_ = 3.78-19.16 μM) [144]. A series of osthole derivatives (an ingredient of the traditional Chinese medicine extracted from *Cnidium monnieri* (L.) Cusson), bearing aryl substituents at position 3 of the coumarin, have been prepared and evaluated for their growth inhibitory activity against human breast cancer MCF-7 and MDA-MB-231 cell lines. Amongst them, compound **61** has been found to be the most potent molecule, with IC_50_ values of 0.24 and 0.31 μM against MCF-7 and MDA-MB-231, respectively, improving more than 100-fold the values of osthole [145]. Coumarin-monastrol hybrids (compound **62**) have also been designed and synthetized, and further evaluated against both hormone receptor (positive and negative) breast cancers, which selectively induce apoptosis in both primary and metastatic breast cancer cell lines [146]. Steroids 3-*O*-sulfamates are known as powerful steroid sulphatase (STS) inhibitors. STX64 (667 Coumate, Irosustat), a tricyclic coumarin-based sulfamate that irreversibly inhibits STS activity, has been selected to enter into phase I clinical trials as an STS inhibitor for postmenopausal women with breast cancer [147]. Inspired by these data, several series of 3-phenylcoumarin-7-*O*-sulphamate derivatives have been further studied. Fluorinated-, 4-aminophenyl-*N*-phosphorylated- and *N*-thiophosphorylated-7-*O*-sulphamate-3-arylcoumarins (compounds **63**, **64** and **65** respectively) have been studied by the same research group, showing more than 10 times higher inhibitory potency than coumarin-7-*O*-sulfamate as inhibitors of STS. Amongst the studied compounds, the most interesting proved to be compound **63**, both displaying IC_50_ values of 0.27 μM [148], a similar IC_50_ of approximately 0.20 μM for the phosphorylated derivatives (compounds **64** and **65**) [149,150]. Supported by molecular modelling techniques, fluorinated compound **66** has been designed, synthetized and then tested, proving to display an IC_50_ of 0.18 μM against STS, and an IC_50_ of 15.9 μM and 8.7 μM, respectively, against MCF-7 and hypotriploid human cells (T47D) [151]. These values are in the same range of tamoxifen IC_50_ = 6.8 and 10.6 μM, respectively. 3-Phenylcoumarins substituted by simple groups also proved to inhibit the 17-β-hydroxysteroid dehydrogenase 1, an enzyme of the last steps of the sulfatase pathway (values between > 68% of inhibition and IC_50_ = 1 μM), and only modestly activates with 17-β-hydroxysteroid dehydrogenase 2, which is interesting for lowering the effect of estradiol levels in vivo. In the studied series, the substitution of the 3-phenyl ring with the 3-imidazole ring in the coumarin scaffold assures strong and selective aromatase inhibition [152].

### 5.3. Other Pharmacological Interests

In this section, all the relevant references found for those pharmacological interests that do not fit clearly into the previous sections are included. Even so, these activities have also been grouped in the most convenient way and subdivided into the following sub-sections.

#### 5.3.1. Enzymatic Inhibitors

**Figure 22:**
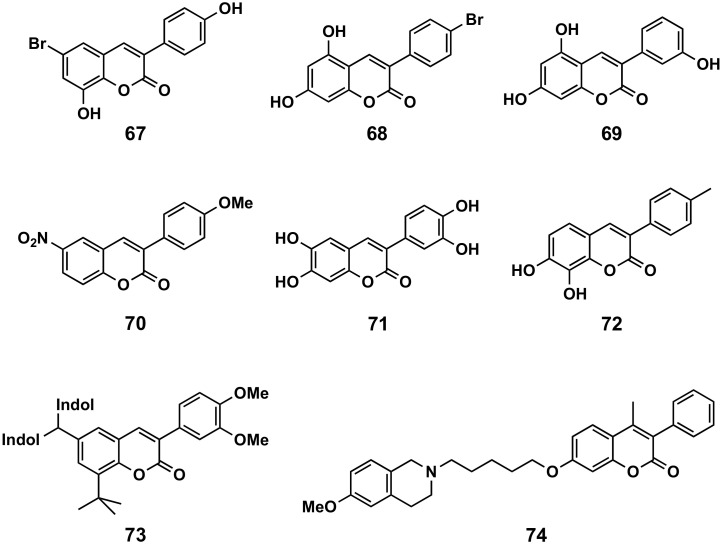
3-Phenylcoumarins as enzymatic inhibitors: tyrosinase (**67** and **68**), xanthine oxidase (**69** and **70**), glutathione S-transferase (**71**), HMG-CoA reductase (**72** and **73**) and P-glycoprotein (**74**).

Different enzymes and proteins involved in different physiologic pathways, and therefore in different pathologies, have been studied. Tyrosinase catalyzes the first step of melanogenesis, a physiological pathway involved in the formation of melanin. From the first studied series of bromohydroxycoumarins, compound **67** (Figure 22) proved to be the best mushroom tyrosinase inhibitor, displaying an IC_50_ of 215 μM, being better than umbelliferone [153]. This activity has been improved by the dihydroxybromo analogue **68** (Figure 22), with an IC_50_ of 0.19 μM, approximately 100 times more active than kojic acid (IC_50_ = 17.9 μM) [154].

Xanthine oxidase is a metalloenzyme whose main function is to hydroxylate a series of substrates, and constitutes an interesting target for the hyperuricemia and synergic treatment of several diseases. Among all the evaluated candidates, compound **69** (Figure 22) proved to be the best inhibitor, with an IC_50_ of 2.13 μM, being 7-fold better than allopurinol, the reference compound (IC_50_ = 14.75 μM). Compound **69** proved to be non-cytotoxic at its IC_50_ value, as demonstrated by the viability of 99% in T3 cells [155]. Compound **70** (Figure 22) shows an IC_50_ value of 8.4 pM [156].

Glutathione S-transferase plays an important role in detoxification by catalyzing the conjugation of many hydrophobic and electrophilic compounds, with reduced glutathione. A series of compounds has been evaluated, with compound **71** (Figure 22) being the most active, which displaying an IC_50_ of 13.5 μM [157].

3-Hydroxy-3-methylglutaryl-CoA (HMG-CoA) reductase inhibition-lipid-lowering is a control enzyme of the mevalonate pathway, involved in the production of cholesterol and other isoprenoids. A series of simple 3-phenylcoumarins have been evaluated, and compound **72** (Figure 22) displayed the highest HMG-CoA reductase inhibitory activity in vitro (IC_50_ = 42 μM) [158]. Also, a series of coumarin-indole hybrids have been synthesized and evaluated in an in vitro model of the HMG-CoA reductase enzyme. Among the hybrids, compound **73** (Figure 22) proved to be the best, as it significantly reduced the serum and hepatic lipid profiles in an HFD-fed hyperlipidemic rat model, being the mechanism of action associated with the regulation of HMG-CoA reductase activity in the liver [159].

P-glycoprotein is an important protein of the cell membrane that pumps many foreign substances out of cells. Therefore, they could be interesting, for example, in co-administration with other drugs to increase their duration of action. A series of conjugates bearing a 1,2,3,4-tetrahydroisoquinoline motif linked to substituted 7-hydroxy-3-phenylcoumarin has been assayed, obtaining structure–activity relationships according to the nature and length of the different substituents, and obtaining compound **74** (Figure 22) with an IC_50_ of 0.22 μM in the multidrug resistance protein 1 (MDR1) [160].

#### 5.3.2. Global Pharmacological Effects

Glycycoumarin (Figure 2), a representative coumarin compound in liquorice, as well as an edible and medicinal plant, has long been used to treat different diseases, including liver diseases and hepatic disorders. A hepatoprotective effect of glycycoumarin has been reported, with glycycoumarin being highly effective against alcoholic liver disease, non-alcoholic fatty liver disease, acetaminophen-induced hepatotoxicity and liver cancer through mechanisms involved in activation of the Nrf2 antioxidant system, stimulation of AMPK-mediated energy homeostasis, induction of autophagy degradation process, and inhibiting oncogenic kinase T-lymphokine-activated killer cell-originated protein kinase activity [8].

**Figure 23:**
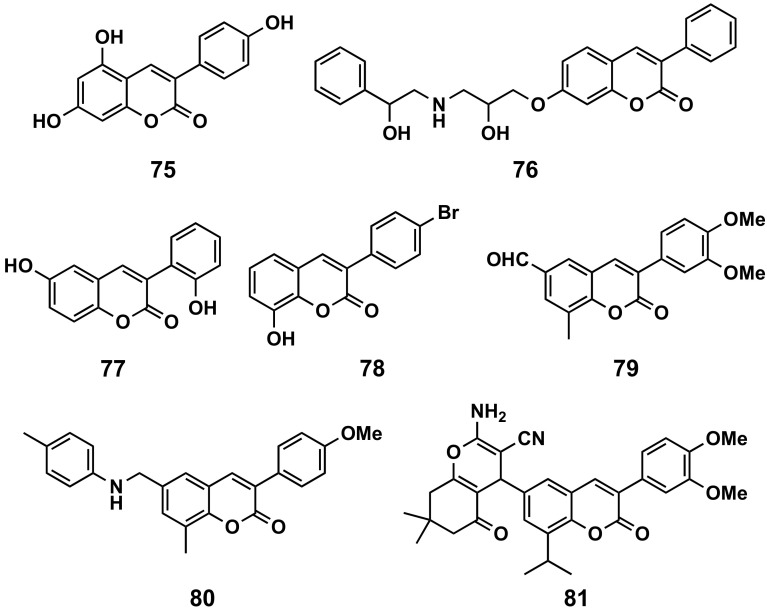
3-Phenylcoumarins presenting other global pharmacological effects: anti-diabetic agents (**75** and **76**), antiallergic (**77**), adenosine ligand (**78**) and antidepressants (**79**–**81**).

A variety of 3-arylcoumarin derivatives have been screened for their antioxidant, α-glucosidase inhibitory and advanced glycation end-products (AGEs) formation inhibitory activities. Most of the described compounds exhibited significant antioxidant and AGEs formation inhibitory activities, highlighting the trihydroxy derivative **75** (Figure 23, α-glucosidase inhibitory activity, IC_50_ = 1.37 μM; AGE formation inhibitory activity, IC_50_ = 2.52 μM), showing that phenyl at position 3 of the coumarin offers better results than the phenyl at position 4. Anti-diabetic activity studies showed that some compounds are equipotent to the drug glibenclamide in vivo [36]. Glycycoumarin (Figure 2), isolated from the roots and rhizomes of liquorice (Glycyrrhiza uralensis), inhibited α-glucosidase inhibitory activity, with IC_50_ values of 0.1 μM [161]. From a medicinal plant, Selaginella rolandi-principis, a new coumarin, selaginolide A (Table 1), has been isolated and investigated in vitro on the 2-(*N*-(7-nitrobenz-2-oxa-1,3-diazol-4-yl)amino)-2-deoxyglucose (2-NBDG) uptake in 3T3-L1 adipocytes and against protein-tyrosine phosphatase 1B (PTP1B) and α-glucosidase enzyme activities as well. This compound exhibited high potency with inhibitory IC_50_ values of 7.40 and 7.52 μM against PTP1B and α-glucosidase, respectively, which makes it a potential starting point for the development of antidiabetic agents [6]. In vivo studies against streptozotocin (STZ)-induced diabetes in rats have been performed with a series of 3-phenylcoumarins substituted at position 7 by a complex ether chain. Several compounds proved to have a good profile, with compound **76** (Figure 23) being the most interesting, comparable with glibenclamide (IC_50_ = 0.22 μM, MDR1) [162].

The dyhydroxy derivative **77** (Figure 23) turned out to be interesting as an antiallergic [163], after a study among a series of 3-arylcoumarin that have been evaluated as inhibitors of mast cell degranulation. This was done based on their close structural similarity to flavonoids, whose anti-allergic activity has been extensively reported.

Adenosine receptors are involved in several physiological processes. Some publications refer to the study of the binding activity and selectivity of this type of compounds on adenosine receptors. Synthetic compounds have been evaluated by radioligand binding (A_1_, A_2A_ and A_3_) and adenylyl cyclase activity (A_2B_) assays in order to study their affinity for the four human AR subtypes. None of the studied compounds showed affinity for the A_2B_ receptor, while quite a few compounds have been found to be nonselective ligands. Various ligands studied proved to be selective to the A_3_ receptor, with compound **78** (Figure 23) being the most potent (K*_i_* = 258 nM) [164,165,166].

Several compounds of this class exhibited impressive antidepressant activity, measured in terms of duration and percentage of decrease in the immobility duration. A low dose of 0.5 mg/kg of compound **79** (Figure 23) significantly decreased the immobility time, exhibited greater efficacy than the fluoxetine and imipramine and did not show any neurotoxicity in the rotarod test [167]. Compound **80** (Figure 23) showed antidepressant-like behavior in the mouse forced swim test (FST) at low dose of 0.5 mg/kg (80%) intraperitoneal, to a greater extent than the standard drug fluoxetine at a dose of 20 mg/kg (70%) [168]. Compound **81** (Figure 23) showed antidepressant-like behavior in the mouse FST at a low dose of 0.5 mg/kg (86%) intraperitoneal, to a greater extent than the standard drug fluoxetine at a dose of 20 mg/kg (70%) [169].

## 6. Other Interests: Fluorescent Probes

The 3-phenylcoumarins are a privileged scaffold not only for their interesting and varied specific pharmacological activities, but also for other physicochemical properties and, in particular, for their potential as fluorescence probes. Due to the interesting and large number of studies that exist on this in recent years, they deserve an independent review. In the current overview, very succinct examples have been selected which are considered the most significant. Fluorescent biosensors have been developed to enable imaging and monitoring of a variety of metabolites and cellular events. This can be done by direct visualization and analysis; however, 3-phenylcoumarins also offer enormous possibilities in biorthogonal fluorogenic reactions, which allow not only the visualization of a fixed situation but also, in many cases, to follow the transformations throughout complex metabolic processes [170,171]. These compounds can be useful as fluorescent biosensors for the detection of hydroxyl radicals, and therefore can be potentially applied in the diagnosis of oxidative stress in the human body [172], metal cations such as Fe^3+^ [173] or anions such as carbonate [174]. 3-Phenylcoumarins can be used to analyze and study different biochemical compounds such as flavin [175,176] or anatomical/physiological states, such as the state of neuronal myelination [177], histamine released by mast cells [178], access to mitochondria [179] and others, which can be identified very directly with some metabolic problem, which in turn can be linked to a disease or disorder. On occasion, this may allow or facilitate the study of new pharmacological agents in oxidation/reduction processes [180], but also in more specific processes such as estrogen receptors and breast cancer [132], bioinorganic anticancer compounds [181], MAOs [182], etc. For these functions, they can also be associated with other groups or molecules, as there are examples with fluorenes or xanthenes [183], tetrazines [184], rhodamine [185] or with different polymers [186].

## 7. Conclusions

3-Phenylcoumarins proved to be very promising molecules, both in organic and medicinal chemistry. Their potential has been described in this review based on more than 180 papers published in the past decade. Their chemical and physicochemical properties allow for a huge range of applications for this family of compounds. Six new natural phenylcoumarins were isolated in this period, and better synthetic methods have been applied to obtain this scaffold. Depending on their substitution patterns, the interest of less specific biological properties such as its antioxidant capacity, as well as those more specific, such as interaction with enzymes and/or receptors, convert the 3-phenylcoumarin scaffold into a privileged substructure from the pharmacological point of view. The pharmacological fields most explored in the past decade have been neurodegenerative disorders, especially Alzheimer’s or Parkinson’s diseases, due to interaction with AChE and MAO-B, respectively, but also including effects on inflammation or on the cardiovascular system, and in the field of chemotherapeutic agents, with special attention to different types of cancer. Other pharmacological interests have been less explored so far. Finally, the interest of 3-phenylcoumarins is growing in chemical biology, as they are being extensively used as fluorescent probes.

## Figures and Tables

**Table 1:** Naturally-occurring 3-phenylcoumarins identified in the past decade.

Compound	Origin	Reference
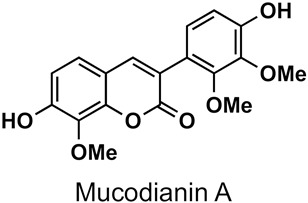	*Mucuna birdwoodiana*	[1]
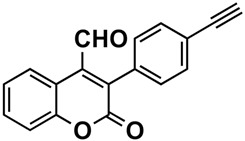	*Tetramorium* sp.	[2]
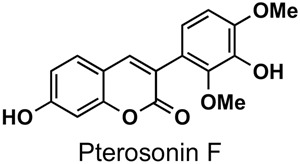	*Pterocarpus soyauxii*	[3]
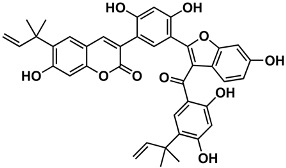	*Sphenostylis marginata*	[4]
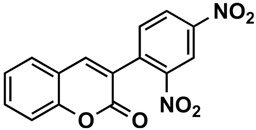	*Rhizophora mucronata*	[5]
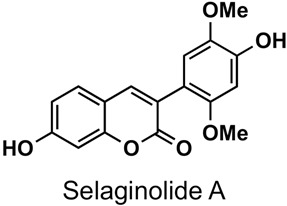	*Selaginella rolandi-principis*	[6]

## Data Availability

Not applicable.
